# Cross-Regional Elemental Comparison of Mussels Using Total Reflection X-Ray Fluorescence (TXRF)

**DOI:** 10.3390/molecules30020283

**Published:** 2025-01-13

**Authors:** Nina-Nicoleta Lazăr, Ira-Adeline Simionov, Mădălina Călmuc, Valentina-Andreea Călmuc, Cătălina Iticescu, Puiu-Lucian Georgescu, Mihaela Timofti, Silvia Drăgan

**Affiliations:** 1 REXDAN Research Infrastructure, ‘Dunărea de Jos’ University of Galati, 98 George Coșbuc Street, 800385 Galati, Romania; nina.condurache@ugal.ro (N.-N.L.); ira.simionov@ugal.ro (I.-A.S.); madalina.calmuc@ugal.ro (M.C.); valentinacalmuc@yahoo.ro (V.-A.C.); catalina.iticescu@ugal.ro (C.I.); 2 Faculty of Food Science and Engineering, ‘Dunărea de Jos’ University of Galaţi, 47 Domnească Street, 800008 Galați, Romania; 3 Faculty of Sciences and Environment, ‘Dunarea de Jos’ University of Galati, 111 Domnească Street, 800008 Galati, Romania; 4 Enviro Ecosmart SME, 189 Tecuci Street, 800552 Galati, Romania; silvia.dragan@gmail.com

**Keywords:** TXRF, chemical elements, *Mythilus galloprovincialis*, *Mythilus chilensis*, aquaculture, cross-regional comparison

## Abstract

This study evaluates the effectiveness of Total Reflection X-ray Fluorescence for multi-element analysis in mussels, focusing on sensitivity, precision, and detection limits. Additionally, it offers a cross-regional comparison of elemental composition in mussels from aquaculture farms in Italy, Spain, and Chile. TXRF, using suspensions of mussel samples, proved effective in detecting minor and trace elements, with recovery rates over 80% for Fe, Cu, Zn, As, and Sr. The research offers a chemical element comparison of *Mytilus galloprovincialis* and *Mytilus chilensis* mussels, revealing significant variation based on geographic origin. Correlation matrices demonstrated variable associations between elements, indicating that regional environmental conditions influence bioaccumulation. These findings deepen our understanding of how mussels accumulate elements in different environments. However, further research is needed to develop comprehensive elemental databases and to account for seasonal and temporal variations in mussels’ elemental composition. This study may bring insight for food safety and public health monitoring.

## 1. Introduction

Mussels are a popular type of seafood consumed worldwide. In terms of production, Europe holds a significant position, contributing to over a third of the overall global yield. Notably, only 20% of this quantity comes from fishing, with the remaining 80% originating from aquaculture [1]. Among the primary species harvested and cultivated in Europe, *Mytilus galloprovincialis* (Lamarck, 1819) and *Mytilus chilensis* (Hupé, 1854) stand out as key contributors [2]. According to the FAO, Spain, France, and Italy account for two-thirds of the total mussel production and 78% of the total consumption in Europe [2].

The cultivation process of mussels can significantly impact their elemental composition due to factors like environmental exposure, feeding habits, and water quality, which vary [3]. Certain non-essential elements can accumulate to toxic levels, posing serious health risks through human consumption. Consequently, many countries have established seafood safety regulations [4,5]. Despite these concerns, mussels also provide essential nutrients, making it important to understand their elemental profile for both safety and nutritional value.

Reliable analytical methods are needed to accurately assess nutritional composition and detect trace pollutants. Techniques like Inductively Coupled Plasma Spectrometry (ICP-MS/ICP-AES) and Atomic Absorption Spectroscopy (AAS) are generally favored for their accuracy and low detection limits. However, both types of techniques have lots of disadvantages and even limitations. Firstly, both techniques require additional sample treatment, such as acidic digestion, which can lead to analyte losses and is also time- and money-consuming [6,7]. The ICP techniques have high operation costs, as they require argon to produce the plasma that converts the sample into ions that are then measured. For certain elements’ analysis, multiple high-purity gases may be required [8]. AAS techniques have a limited analytical range and, depending on the type of AAS, may only be suitable for single-element analysis, which can be time-consuming [8]. Therefore, alternative, cost-effective methods with high accuracy are being sought.

Total Reflectance X-ray Fluorescence (TXRF) emerges as a promising tool, offering high sensitivity, multi-element capability, and minimal sample preparation, and it is expected to be further utilized in scientific research [9]. TXRF allows solid samples to be directly analyzed by suspensions and does not require gases or expensive reagents, thus eliminating high costs. It has a high sensitivity and low detection limits down to the ppb level [3,10]. It is a non-destructive, micro-analytical technique suitable for analyzing small sample quantities [10,11,12]. Surprisingly, in the specialized literature, very few published studies have analyzed the elemental concentration of mussels using the TXRF technique [13,14,15,16]. Despite their disadvantages, most researchers prefer and utilize the previously mentioned methods.

This study aims to assess the performance of TXRF for multi-element analysis in mussels, with two primary objectives:

- Evaluate TXRF’s effectiveness, focusing on sensitivity, precision, detection, and quantitation limits for various elements in mussel tissues.
- Characterize the elemental profiles of mussels from three different aquaculture farms regarding nutrients and contaminants and analyze the correlation between them.

The novelty of the study stands in offering the first cross-regional elemental comparison of mussels (*M. galloprovincialis* and *M. chilensis*) from Italian, Spanish, and Chilean aquaculture using the TXRF technique. The specialized literature typically focuses on comparisons of mussels from the same region [17,18] or from different regions [19,20,21], but not from the regions considered in our study, and none utilize TXRF for elemental assessment.

## 2. Results and Discussion

### 2.1. TXRF Method Validation

Method validation is an important step in analytical chemistry that ensures a technique is suitable for its intended purpose. It involves systematic evaluation of the performance parameters of the analytical technique to assure its suitability for application [22,23]. TXRF is an accurate method utilized for multi-element analysis of various samples, including matrices, such as mussels.

**Figure 1:**
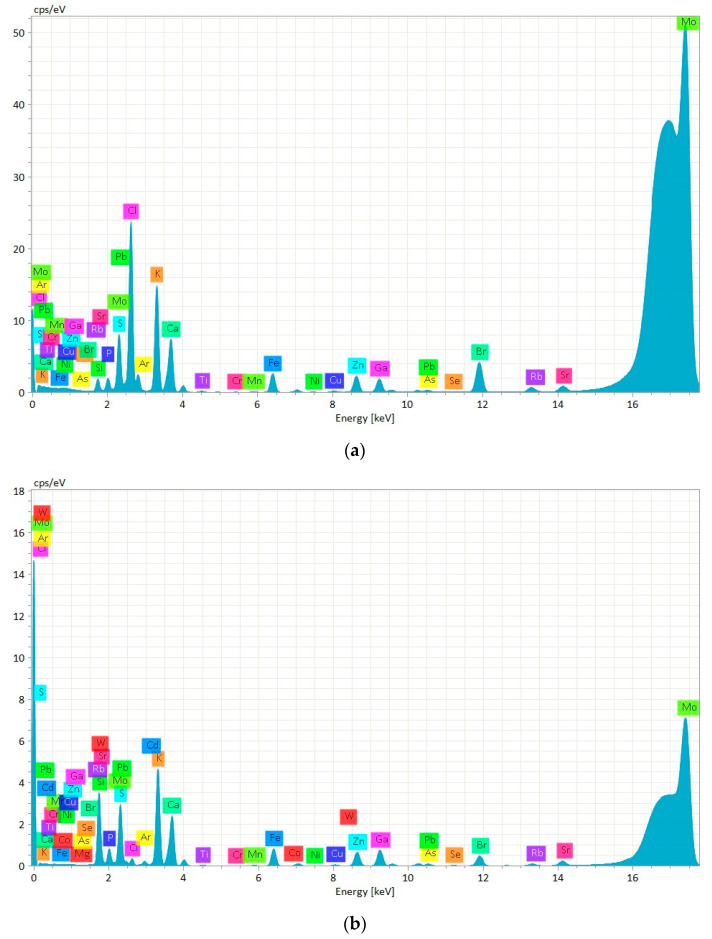
Chemical elements’ spectra of suspension (**a**) and digestion (**b**) in mussels CRM.

In our study, with the aid of the TXRF technique, we successfully identified elements, such as P, S, Cl, K, Ca, Ti, Cr, Mn, Fe, Ni, Cu, Zn, As, Se, Br, Rb, Sr, and Pb, in both suspended and digested mussels CRM, as shown in Figure 1. Except for Pb, which was identified and quantified using the Lα emission lines at 10.551 keV, all other elements were detected using their specific Kα emission lines (Figure 1). Before CRM, blank analyses were conducted by measuring 10 quartz discs prepared in the same way as the CRM discs.

In the spectra of both samples can also be seen elements like silicon (Si), argon (Ar), and molybdenum (Mo) (Figure 1). The Si peak was attributed to the quartz sample carrier, while the Ar was observed due to its natural occurrence in the atmosphere. The presence of Mo was justified by its role as an anode element in the X-ray tube of the TXRF equipment. These three elements were always present in the spectra of the samples due to the analysis conditions.

**Figure 2:**
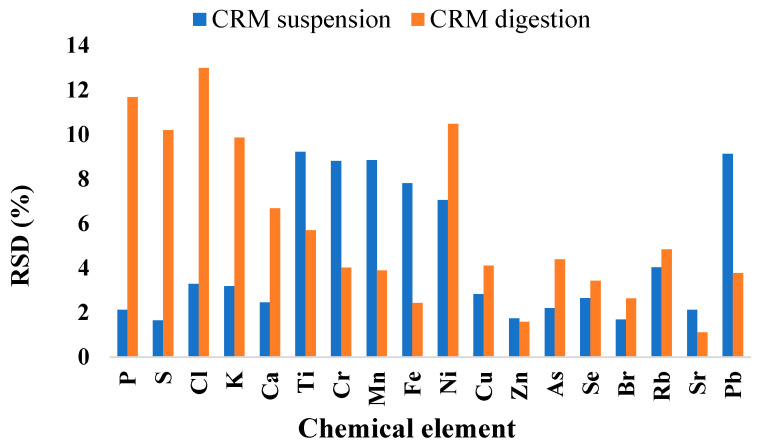
Relative standard deviation (RSD%) for TXRF analysis of suspended and digested mussels CRM.

Method validation ensures that the TXRF approach produces precise and reliable results, offering confidence in the data provided by its application. In this study, parameters, such as LLD, LOQ, RSD, and R%, were assessed during method validation. The results of each parameter are presented in Table 1 and Table 2 and Figure 2.

Using a CRM suspension resulted in significantly higher concentrations (*p* < 0.05) according to Table 1 and, hence, significantly higher recovery rates (Table 2) for most detected elements compared to the digested CRM. Several investigations have shown that there can be a significant loss of specific chemical elements due to the potential formation of precipitates containing trace elements during the digestion of biological materials [24,25]. Our results comply with those of Margui et al. [15] and Nurhaini et al. [26], who also observed higher element concentration in suspended rather than digested CRM. However, in our study, the Pb showed a higher concentration in digested rather than suspended CRM. Likewise, Ishak et al. [27] reported that wet digestion using HNO_3_ and H_2_O_2_ was the most effective method for achieving high precision, accuracy, and recovery for Mn and Mg. Furthermore, none of our samples exhibited detectable levels of elements like Cd, Hg, and Co, most likely due to concentrations below the TXRF’s detection limit or high volatility in the Hg case. Moreover, elements like Mg and Na were not detected (Table 1), most likely due to the Mo tube’s limitations concerning low-energy elements.

Considering the low LLD and LOQ values presented in Table 1, we can appreciate that each element was detected and quantified with certainty. Our results are in accordance with those reported by Beltrán et al. [28]. These results indicate that the LLD of various elements was below 1 ppm (µg/g), confirming that TXRF exhibits high sensitivity and can detect elements down to the ppb (ng/g) level.

From Table 2, it can be observed that an R% between 38.60 ± 1.27% and 179.59 ± 15.82% was achieved for the suspension of CRM, whilst for the digested one an R% between 1.32 ± 0.17% and 152.06 ± 6.98% was obtained. In both cases, the lowest R% values were obtained for the Cl, which is because this chemical element is highly volatile and the mineralization step accelerates the process of volatilization.

In the case of Cr and Ni for both types of samples, the R% showed higher values than 100% (Table 2), indicating possible contamination prior to analysis. Particularly good recovery rates were achieved when using the suspension of the CRM for Fe, Cu, Zn, As, and Sr, with values higher than 89%. According to AOAC [29], the expected R% as a function of analyte concentration is 80–110% for a 1 to 10 mg/kg analyte and 90–107% for a 100 mg/kg analyte. In addition, optimized extraction methodologies are necessary to achieve maximum recovery rates for K, Ca, Mn, Se, Rb, and even Pb.

The RSD gives the precision of the set of values obtained, which is also an important parameter for validating an analytical method. In our study, the RSD results are illustrated in Figure 2.

From Figure 2, it can be observed that the best RSD, with values below 10%, was obtained for the analysis of CRM in suspension compared to the digested CRM. A notable RSD was observed for elements including P, S, Cl, K, Ca, Cu, Zn, As, Se, Br, Rb, and Sr, all exhibiting values below 5% when measured in the suspended CRM. RSD values for the suspension of CRM are in accordance with AOAC [29], who indicated that a maximum value of 11% for a concentration of 1 mg/kg analyte, 7.3% for 10 mg/kg analyte, and 5.3% for 100 mg/kg analyte is preferable. Therefore, it can be claimed that the proposed method showed reasonable precision based on the obtained RSD values.

Consequently, using the TXRF technique with a suspension of the mussel samples is a promising, easy, fast, and low-cost method for identifying minor and trace elements. Therefore, suspensions were used further in the study of *M. galloprovincialis* and *M. chilensis* from multiple sources.

### 2.2. Elemental Composition of Mussels

**Figure 3:**
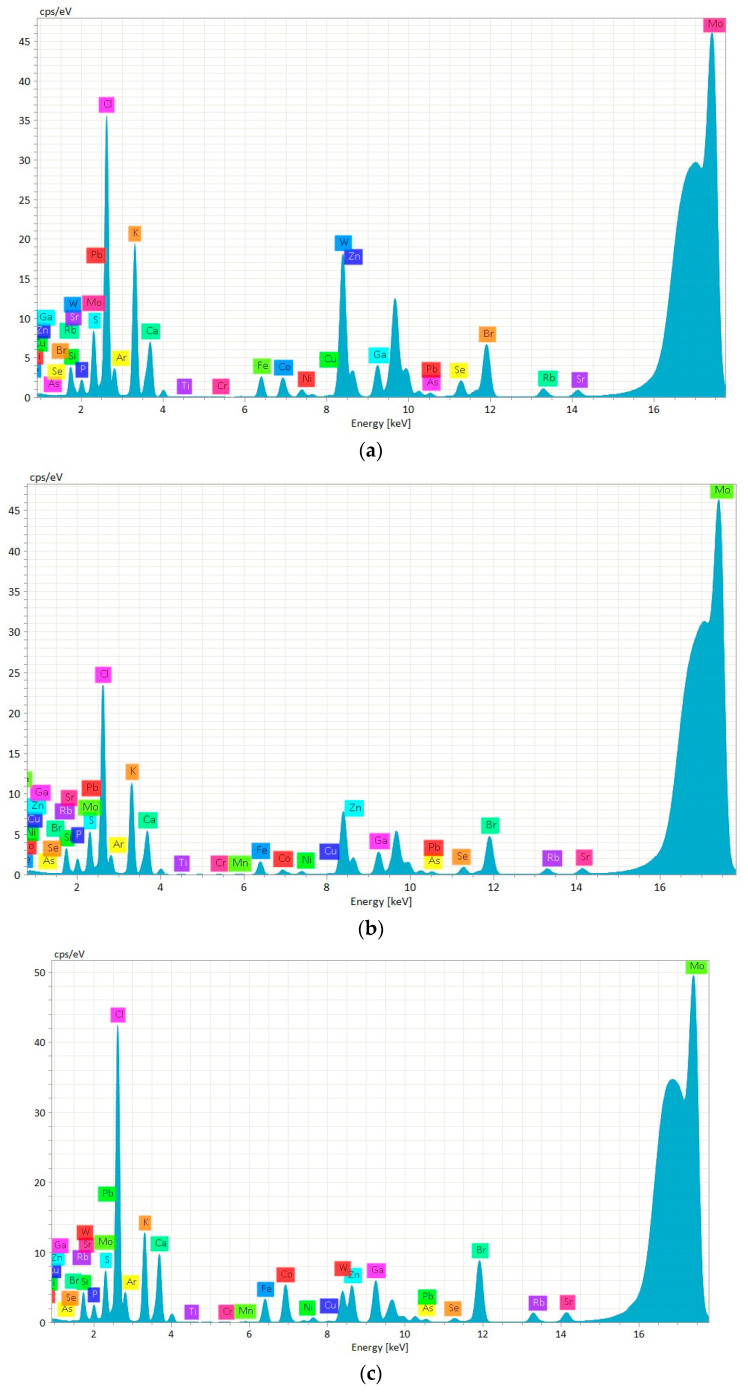
Chemical elements identified in *M. galloprovincialis* mussels from Italy (**a**) and Spain (**c**) and *M. chilensis* mussels from Chile (**b**).

The TXRF technique, renowned for its high sensitivity and multi-element capability, was employed in this study for the precise detection and quantification of various elements present in the mussel samples. Figure 3 shows the chemical elements identified in the aquaculture mussels’ specimens using the technique mentioned above. The spectra images display the elements found in mussels from three different regions: Italy, Spain, and Chile. In all specimens, elements, such as P, S, Cl, K, Ca, Ti, Cr, Mn, Fe, Co, Ni, Cu, Zn, As, Se, Br, Rb, Sr, and Pb, were identified using TXRF (Figure 3). In addition, tungsten (W) was identified in all of the samples, which is attributed to the use of a tungsten mill for grinding the mussels. However, W, along with Si, Ar, and Mo, were deconvoluted to eliminate any potential interferences in the concentration calculations.

Our results comply with other studies. For example, Bohuss et al. [13] also identified Ca, K, Sr, Cu, Fe, Zn, Ni, Mn, and Pb in digested zebra mussels using the TXRF technique. Margui et al. [15] identified and quantified elements like Mn, Fe, Cu, Zn, As, Se, Sr, and Cd in both digested and suspension clams using the TXRF technique. Elements, such as P, S, Cl, K, Ca, Mn, Fe, Co, Ni, Cu, Zn, Se, Br, Rb, and Sr, were also identified in the Vietnamese mussel muscle using the TXRF technique earlier by Brauer et al. [14]. In 2004, Wagner and Boman [16] reported As, Ba, Be, Br, Ca, Cd, Cr, Cu, Fe, K, Mn, Ni, P, Pb, Rb, S, Se, Sr, Ti, and Zn in the Vietnamese freshwater mussels analyzed with the TXRF technique.

Based on the identified elements and the results from the method validation, only the chemicals achieving a recovery rate (R%) higher than 80% were further considered. This threshold ensured the reliability and accuracy of the quantification process. Consequently, only Fe, Cu, Zn, As, and Sr were quantified in our study using the TXRF method. Our findings are consistent with other studies. For instance, Marguí et al. [15] concluded that after evaluating TXRF, ICP-MS, and ED-XRF for the analysis of chemical elements in edible clams, the TXRF technique was more suitable for detecting Ti, Mn, Fe, Zn, Br, and Sr than the other techniques considering the method validation results. In the specialized literature, the TXRF technique was also successfully used on other types of samples. For instance, Lossow et al. [30] reported a strong correlation between the levels of Fe, Cu, Zn, and Se measured through TXRF and those quantified through ICP-MS in murine liver tissue samples. A strong correlation between the concentrations of iodine (I) from dietary supplement products obtained with both TXRF and ICP-AES was also reported by Varga [31].

Table 3 presents the detailed results derived from the TXRF analysis, showing the concentrations of individual elements detected within the mussel samples. The obtained values were corrected for the water content and expressed as dry weight (DW).

Table 3 highlights that the Fe concentration ranged from 0.46 ± 0.01 g/kg DW in M.C.C.2 to 1.99 ± 0.08 g/kg DW in M.G.S.3. Spanish mussels’ samples exhibited higher Fe levels than the Chilean samples. Cu levels varied significantly, with the highest in M.C.C.3 (34.03 ± 0.49 mg/kg) and the lowest in M.G.I.2 (7.84 ± 0.14 mg/kg DW). *M. chilensis* samples tended to have higher Cu concentrations. The Zn concentration ranges from 0.41 ± 0.02 g/kg DW in M.C.C.2 to 2.31 ± 0.02 g/kg DW in M.G.I.3. Italian samples generally showed higher Zn levels compared to the other regions. The highest As levels were found in M.G.I.3 (87.42 ± 1.12 mg/kg DW) and the lowest in M.G.S.3 (27.42 ± 1.62 mg/kg DW). Italian samples exhibited higher arsenic levels overall, as shown in Table 3. The highest Sr concentrations were in M.G.I.3 (238.63 ± 4.12 mg/kg DW), and the lowest were in M.C.C.2 (75.06 ± 2.32 mg/kg DW). Sr levels were generally higher in *M. galloprovincialis* compared to *M. chilensis*.

All of these data show a considerable variation in the elemental composition of mussels depending on the rearing regions. Notably, *M. galloprovincialis* from Italy showed higher concentrations of most elements compared to samples from Spain and Chile. This variation might be due to geographical variation, which leads to differences in water chemistry, pollution levels, or aquaculture practices.

In terms of aquaculture practices, the method of mussel cultivation significantly influences their elemental profile due to variations in environmental exposure, feeding patterns, and water quality. Different mussel cultivation methods are applied globally, depending on regional and local environmental conditions [32,33]. Italy is known to use the rope culture. In this method, mussels are grown on ropes suspended in the water column, either in sheltered bays or offshore [34]. Mussels grown on ropes can access a diverse range of plankton and nutrients, leading to a varied elemental profile that may be richer in certain minerals and trace elements [35]. Spain is known for its extensive use of raft culture, particularly in the Galician region [36,37]. Mussels grown on rafts in these nutrient-rich waters often experience enhanced growth and an improved elemental composition. Their limited interaction with the seabed can reduce the uptake of certain elements found in sediments, potentially resulting in a cleaner elemental profile. Another type of cultivation is longline culture, used in various parts of Europe and Chile [38]. Mussels are grown on long ropes suspended in the water and supported by buoys. Mussels cultivated in the open sea are exposed to different water currents and nutrient flows, which can influence their elemental uptake. The open-sea environment often means lower exposure to coastal pollutants [39]. The depth at which the mussels are suspended can affect their access to different nutrients and elements, impacting their overall elemental profile [38]

Other aquaculture practices can also influence the mussel elements’ profile. For instance, the high Cu concentrations in mussels could signal the use of copper-based treatments for controlling biofouling in aquaculture systems [40,41,42]. In addition, the high concentrations of Fe and Zn in the mussels from Italy and Spain may be the result of water sediment disturbance or fertilizer runoff [43,44,45].

The quality of water, including its temperature, salinity, and pollution levels, also plays a significant role. Because mussels filter large volumes of water, any contaminants or nutrients present can be absorbed and reflected in their chemical profile [46]. Usually, mussel farms are located in nutrient-rich coastal locations with river runoff, which fosters high primary production and fast mussel development [47]. Thus, any modifications in water composition due to pollution or seasonal or climate changes are easily reflected in the mussels’ composition.

### 2.3. Chemical Elements’ Correlation

**Figure 4:**
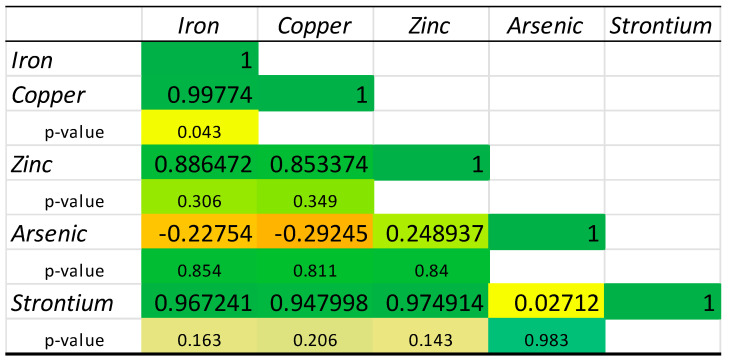
Correlation matrix of the chemical elements quantified in the mussels from Chilean aquaculture. Red color—lowest value, yellow color—average value, green color—highest value

**Figure 5:**
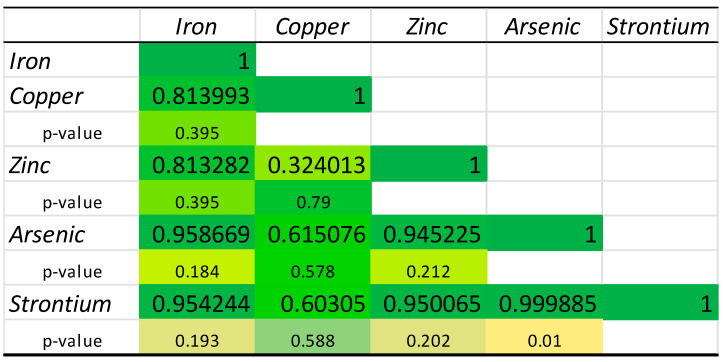
Correlation matrix of the chemical elements quantified in the mussels from Italian aquaculture. Red color—lowest value, yellow color—average value, green color—highest value

**Figure 6:**
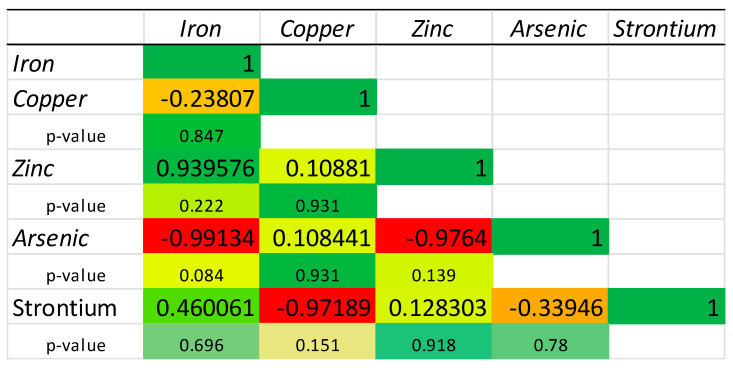
Correlation matrix of the chemical elements quantified in the mussels from Spanish aquaculture. Red color—lowest value, yellow color—average value, green color—highest value

In our study, high-precision correlation analyses of the chemical elements identified in mussels from each source of origin were also performed. Figure 4, Figure 5 and Figure 6 illustrate these correlations for the mussels from each respective source.

Figure 4 illustrates the relationship between the Fe, Cu, Zn, As, and Sr in Chilean mussels. The strongest positive correlation can be observed between Cu and Fe (0.997, *p*-value < 0.05). This fact suggests that Fe and Cu have a close relationship, and, in most cases, the increase in Fe concentration in Chilean mussels is associated with a similar increase in Cu concentration. Fe, Cu, Zn, and Sr are highly positively correlated with each other, suggesting that factors influencing the concentrations of one element have a similar effect on the others. However, As has weak or insignificant correlations with the other elements, which could indicate that its accumulation mechanisms or sources differ from those of the other elements.

In the case of Italian mussels, the strongest correlation can be observed between Sr and As (0.999, *p*-value < 0.05), which may strongly suggest interconnected bioaccumulation processes for these elements (Figure 5). Strong correlations can also be noticed between Fe, As, Zn, and Sr. Cu has moderate correlations with the other elements, indicating positive but weaker links compared to those between Fe, Zn, As, and Sr.

From Figure 6, it can be observed that in the case of Spanish mussels, a strong negative correlation was highlighted between As and Fe (−0.991). In addition, strong negative correlations were also highlighted between As and Zn and Sr and Cu. The highly negative and strong relationships between these elements may suggest opposite uptake processes or regulatory mechanisms for these elements. On the other hand, a strong positive correlation can be observed between Zn and Fe (Figure 6). Overall, there is a large variation in the relationships between elements, with both very strong and very weak correlations, suggesting that each item may be influenced by a different set of factors.

The matrices show both strong positive and negative correlations, as well as weak or insignificant ones, indicating that each element’s concentration is influenced by different factors in each type of mussel. The differences in correlation patterns suggest that environmental conditions, biological processes, or both vary significantly between the regions where these mussels are found, affecting how elements are accumulated. These insights can help in understanding the bioaccumulation processes and environmental influences on mussels from different regions. In addition, this study can also serve as a starting point for future research on food fraud analysis by examining the traceability of mussels’ geographic origin. However, further research in this area is necessary, including studies that consider databases containing elemental profiles of mussels from different regions as well as seasonal factors and temporal variations that may affect element concentrations.

## 3. Materials and Methods

### 3.1. Chemicals and Reagents

Gallium (Ga) standard solution was purchased from SCP Science (Canada), Triton X-100, polyvinyl alcohol (PVA), Suprapur nitric acid 65% (HNO_3_), and perhydrol 30% (H_2_O_2_) from Sigma-Aldrich (Germany), and silicone solution in isopropanol from Serva Electrophoresis GmbH (Germany). Mussel tissues’ certified reference material (CRM) was obtained from JRC (Belgium).

### 3.2. Validation of TXRF Method

The mussel CRM was used to validate the TXRF method. To appropriately select the optimal sample preparation technique, a comparative approach was adopted. The suspension of CRM was compared with mineralized CRM.

For the suspension-based sample, 40 mg of CRM was homogenized with approximately 1.95 mL of a 1% Triton X-100 solution, according to the available international standards (ISO TS 18507:2015 and ISO DIS 20289:2018). Subsequently, Ga was added as the internal standard compound until reaching a final concentration of 1000 µg/L.

For the mineralization-based sample, the method described by Simionov et al. [4] was utilized with slight modifications. Briefly, approximately 0.3 g of CRM was homogenized with 9 mL of 65% HNO_3_ and 1 mL of 35% H_2_O_2_. The mineralization process was carried out using an advanced microwave digestion system (Ethos Easy Milestone, Sorisole, Italy). The mineralization parameters were as follows: 35 min of digestion at 210 °C and 1800 W using PTFE-TFM vessels. Following mineralization, the samples were filtrated and subsequently diluted with ultrapure water until a final volume of 50 mL. Next, 1.78 mL of each sample was homogenized with 0.2 mL of PVA solution (0.3 g/L). Additionally, Ga was added as the internal standard until a final concentration of 400 µg/L was achieved. The analysis procedure is detailed in Section 3.4. Total Reflection X-ray Spectrometry Analysis. The quantified elements were K, Ca, Cr, Mn, Fe, Ni, Cu, Zn, As, Se, Rb, Sr, and Pb.

For each type of prepared CRM, 10 measurements were performed to ensure high confidence in the analytical results and good homogeneity and distribution of the samples on the quartz plates. The standard deviation and mean of the obtained values were calculated for each element, along with parameters like the lowest limit of detection (LLD), the limit of quantitation (LOQ), precision (coefficient of variation), and the recovery rate.

The lowest limit of detection (LLD) represents the minimum detectable concentration of an element in the sample [48]. The LLD of each element was calculated according to Equation (1):

LLD = (3Ci · √NBG)/Ni(1)

where Ci = element concentration, Ni = element net count rate, and NBG = area of the background under the fluorescence peak.

The limit of quantitation (LOQ) reflects the lowest concentration of an analyte in a sample that can be determined with a high level of precision and accuracy under the specified test conditions [49]. According to Nurhaini et al. (2023), the LOQ can be calculated using Equation (2), as follows:

LOQ = (10Ci · √NBG)/Ni(2)

Precision was measured using the coefficient of variance (or relative standard deviation, RSD), and it was calculated using Equation (3). Generally, an RSD below 5% is preferred, and it must not exceed 15% [22,26].

(3) $$\mathrm{RSD}\;\left(\%\right)=\mathrm{SD}/\bar{X}\times 100$$

where SD = standard deviation of replicates and $\bar{X}$ = average of replicates.

The recovery rate (R) was used to calculate trueness, which indicates how close the analytical result is to the real value. It shows the proportion of analytes recovered [50]. According to the Association of Official Analytical Chemistry (Horwitz, 2002), the acceptable R for a 1 ppm concentration is 75–120%, and for 10 ppm, it is 80–115% in laboratory validation [26]. It can be calculated using Equation (4).

(4) $$R\;(\%)=X_{\mathrm{test}}/X_{\mathrm{ref}}\times 100$$

where X_test_ = analytical result and X_ref_ = certificate value.

### 3.3. Mussel Tissue Initial Preparation

Mussel specimens were purchased from local markets in Galati County, Romania, and originated from different sources (Spain—rearing and fishing zone FAO 27; Italy—rearing and fishing zone FAO 37.2.1; Chile—rearing and fishing zone unspecified). The samples consisted of the *Mytilus galloprovincialis* and *Mytilus chilensis* species, representing all aquaculture products. Upon acquisition, the biological material was transported to the laboratory within REXDAN Infrastructure at ‘Dunărea de Jos’ University of Galați, Romania. The soft tissues were washed with deionized water to remove any foreign material and then dried at 105 °C until a constant mass was achieved with 13–25% dry weight. Subsequently, the dried mussels were ground in a tungsten ball mill to reduce the particle size and stored in zippered polypropylene bags until analysis.

Three specimens of mussels from each batch were analyzed, and they were coded using the letter ‘M’ (for mussels/*Mytillus*) and ‘G’ or ‘C’ from the scientific name (‘*galloprovincialis*’ or ‘*chilensis*’), followed by the first letter of the country of origin (I for Italy, S for Spain, C for Chile) and the replicate number.

### 3.4. Total Reflection X-Ray Spectrometry Analysis

#### 3.4.1. Mussel Sample Preparation

Quartz glass discs (Bruker, MA, USA) were chosen as sample carriers in this study due to their relatively low background noise, excellent reflectivity, and smooth surface [51]. All samples were prepared as homogeneous solutions, as described earlier, before being transferred to the sample carriers. Ga was selected as the internal standard for this work because it does not exist naturally in the tested samples, and it was added in concentrations between 2000 µg/L and 3500 µg/L. Next, 10 µL of each sample solution was transferred onto the center of the siliconized quartz sample carrier and dried using a hot plate at 50 °C for 5 min.

#### 3.4.2. Instrumentation

TXRF measurements were conducted using a Bruker S4 T-Star spectrometer (Bruker AXS Microanalysis GmbH, Billerica, MA, USA) equipped with a molybdenum X-ray tube (50 W) operating at 50 kV and 1000 µA, a multilayer monochromator, and a silicon drift detector (SDD) with a 60 mm^2^ area and 149 eV energy resolution. The equipment is the property of the REXDAN Research Center of ‘Dunarea de Jos’ University of Galati, Romania. The measurements were performed in triplicate under ambient air conditions for 1000 s each, and the results were reported as mean ± standard deviation of the triplicates (n = 3). Prior to each analysis, the instrument was calibrated according to the manufacturer’s instructions, which included alignment and concentration calibration using a pre-set standard.

#### 3.4.3. Calculation of Elements Concentration

The concentration of elements within the sample was calculated using TEsprit 1.0 software of the TXRF equipment using the equation below, which is based on Ga’s sensitivity and concentration:

(5) $$C_{i}=C_{\mathrm{IS}}\;\cdot \;N_{i}\;\cdot \;S_{\mathrm{IS}}/N_{\mathrm{IS}}\;\cdot \;S_{i}$$

where C_i_ = element concentration, C_IS_ = internal standard (Ga) concentration, N_i_ = element net count rate, N_IS_ = internal standard (Ga) net count rate, S_i_ = element sensitivity factor, and S_IS_ = internal standard (Ga) sensitivity factor.

### 3.5. Statistical Analysis

The statistical analysis of data was conducted using Minitab 17 software. A descriptive analysis was performed by calculating the mean and standard deviation to understand the data distribution. Moreover, variance analysis was conducted to verify the significant differences between data. Briefly, normality and homoscedasticity tests were performed, followed by ANOVA using Tukey’s post hoc test with a 95% confidence interval. The elemental profile of the mussels was analyzed using the correlation matrix.

## 4. Conclusions

This study demonstrates the effectiveness of TXRF as a reliable, cost-effective method for multi-element analysis in mussels. Elements, such as Fe, Cu, Zn, As, and Sr, were identified and quantified with excellent precision and recovery rates. However, certain elements require optimization of extraction methods. Specifically, methods for Cr, Ni, and Pb need refinement due to the health risks associated with excessive exposure to these elements. Additionally, optimized extraction methods are necessary for P, S, Cl, K, Ca, Mn, and Se, as these elements play essential roles in various metabolic processes in the human body and are required in specific amounts.

The cross-regional comparison of *Mytilus galloprovincialis* and *Mytilus chilensis* from aquaculture in Italy, Spain, and Chile revealed significant variability in elemental profiles, with mussels from Italy showing higher concentrations of several elements. These variations are likely influenced by differences in aquaculture practices across regions. The correlation analysis further highlighted that bioaccumulation processes are shaped by both environmental and biological factors, with regional specificity.

Our findings contribute to the understanding of how mussels accumulate elements under varying environmental conditions, offering important insights for aquaculture management and food safety. Future research should focus on building more comprehensive elemental databases and accounting for seasonal and temporal variations in mussel composition to further enhance traceability and risk assessment in mussel farming. Such databases would provide a robust foundation for understanding regional and global patterns of elemental accumulation in mussels, while also considering factors like water temperature, salinity, and plankton availability, which can significantly influence the bioaccumulation of nutrients and potentially harmful elements. TXRF offers high sensitivity for detecting trace elements, making it ideal for quantifying both essential nutrients and toxic elements in mussels. This ensures accurate data for a wide range of elements, including those present in low concentrations. Its ability to simultaneously analyze multiple elements provides a holistic view of the elemental composition of mussels, which is particularly relevant for building databases that capture the full spectrum of nutritional and toxicological elements. Additionally, TXRF is suitable for both raw and processed samples, allowing for the inclusion of mussels in various states (e.g., fresh, dried, or digested) in the database. This versatility supports the creation of a more representative dataset.

## Figures and Tables

**Table 1:** Results of TXRF method validation parameters.

Chemical Element	Certified Value	CRM Suspension			CRM Digested		
		Concentration	LLD	LOQ	Concentration	LLD	LOQ
P (g/kg)	-	1.30 ± 0.03 ^a^	0.003	0.01	1.16 ± 0.14 ^a^	0.003	0.01
S (g/k)	-	3.69 ± 0.06 ^a^	0.002	0.01	3.26 ± 0.33 ^b^	0.002	0.01
Cl (g/kg)	22.8	8.80 ± 0.29 ^a^	0.002	0.01	0.30 ± 0.04 ^b^	0.001	0.004
K (g/kg)	5.37	3.43 ± 0.11 ^a^	0.001	0.003	3.04 ± 0.30 ^b^	0.001	0.002
Ca (g/kg)	1.83	1.31 ± 0.03 ^a^	0.001	0.002	1.19 ± 0.08 ^b^	0.001	0.002
Ti (mg/g)	-	14.30 ± 1.32 ^a^	0.25	0.83	7.63 ± 0.43 ^b^	0.21	0.71
Cr (mg/kg)	0.73 ± 0.22	1.31 ± 0.12 ^a^	0.15	0.48	0.96 ± 0.04 ^b^	0.13	0.42
Mn (mg/kg)	4.88 ± 0.24	3.16 ± 0.28 ^a^	0.11	0.38	3.06 ± 0.12 ^a^	0.10	0.33
Fe (mg/kg)	161 ± 8	143.63 ± 11.22 ^a^	0.10	0.32	131.27 ± 3.19 ^b^	0.08	0.28
Ni (mg/kg)	0.69 ± 0.15	0.90 ± 0.06 ^b^	0.07	0.23	1.08 ± 0.11 ^a^	0.06	0.19
Cu (mg/kg)	5.98 ± 0.27	6.04 ± 0.17 ^a^	0.06	0.20	5.08 ± 0.21 ^b^	0.05	0.17
Zn (mg/kg)	71 ± 4	67.38 ± 1.17 ^a^	0.06	0.19	60.62 ± 0.96 ^b^	0.05	0.15
As (mg/kg)	6.7 ± 0.4	6.35 ± 0.14 ^a^	0.05	0.15	5.20 ± 0.23 ^b^	0.04	0.12
Se (mg/kg)	1.62 ± 0.12	1.01 ± 0.03 ^a^	0.05	0.15	1.04 ± 0.04 ^a^	0.04	0.12
Br (mg/kg)	-	95.06 ± 1.60 ^a^	0.04	0.14	25.70 ± 0.68 ^b^	0.03	0.11
Rb (mg/kg)	2.46 ± 0.16	1.80 ± 0.07 ^a^	0.05	0.17	1.59 ± 0.08 ^b^	0.04	0.13
Sr (mg/kg)	19 ± 1.2	16.08 ± 0.34 ^a^	0.06	0.19	13.79 ± 0.15 ^b^	0.03	0.10
Pb (mg/kg)	2.18 ± 0.18	0.88 ± 0.08 ^b^	0.05	0.16	1.60 ± 0.06 ^a^	0.04	0.13
Cd (mg/kg)	0.33 ± 0.02	ND	-	-	ND	-	-
Hg (mg/kg)	0.07 ± 0.00	ND	-	-	ND	-	-
Co (mg/kg)	0.21	ND	-	-	ND	-	-
Mg (g/kg)	1.51	ND	-	-	ND	-	-
Na (g/kg)	13.9	ND	-	-	ND	-	-

Values from the same line that do not share a letter are statistically different at *p* < 0.05 based on the Tukey method and 95% confidence; CRM—certified reference material; LLD—lower limit of detection; LOQ—limit of quantitation; ND—not detected.

**Table 2:** Recovery rates of the analyzed CRM samples.

Chemical Element	Recovery Rates (R%)	
	CRM Suspension	CRM Digested
Cl (g/kg)	38.60 ± 1.27 ^a^	1.32 ± 0.17 ^b^
K (g/kg)	63.96 ± 2.04 ^a^	56.52 ± 5.58 ^b^
Ca (g/kg)	71.64 ± 1.76 ^a^	65.04 ± 4.35 ^b^
Cr (mg/kg)	179.59 ± 15.82 ^a^	131.68 ± 5.29 ^b^
Mn (mg/kg)	64.70 ± 5.73 ^a^	62.75 ± 2.44 ^a^
Fe (mg/kg)	89.21 ± 6.97 ^a^	81.53 ± 1.98 ^b^
Ni (mg/kg)	130.07 ± 9.18 ^b^	152.06 ± 6.98 ^a^
Cu (mg/kg)	100.95 ± 2.85 ^a^	84.96 ± 3.49 ^b^
Zn (mg/kg)	94.91 ± 1.65 ^a^	85.38 ± 1.36 ^b^
As (mg/kg)	94.83 ± 2.09 ^a^	77.61 ± 3.41 ^b^
Se (mg/kg)	62.32 ± 1.65 ^a^	64.14 ± 2.20 ^a^
Rb (mg/kg)	73.24 ± 2.95 ^a^	64.63 ± 3.13 ^b^
Sr (mg/kg)	84.61 ± 1.79 ^a^	72.60 ± 0.81 ^b^
Pb (mg/kg)	40.52 ± 3.70 ^b^	73.22 ± 2.77 ^a^

Values from the same line that do not share a letter are statistically different at *p* < 0.05 based on the Tukey method and 95% confidence. CRM—certified reference material. Before CRM, blank analyses were conducted by measuring 10 quartz discs prepared in the same way as the CRM discs. The measured values were zero.

**Table 3:** Elemental concentrations in the analyzed muscle tissues.

Sample	Fe (g/kg DW)	Cu (mg/kg DW)	Zn (g/kg DW)	As (mg/kg DW)	Sr (mg/kg DW)
M.G.I.1	1.00 ± 0.07 ^c^	19.52 ± 0.30 ^c^	0.59 ± 0.03 ^e^	69.26 ± 0.81 ^b^	120.00 ± 2.13 ^e^
M.G.I.2	0.71 ± 0.07 ^d^	7.84 ± 0.14 ^d^	0.99 ± 0.00 ^c^	66.80 ± 3.97 ^b^	106.33 ± 0.46 ^f^
M.G.I.3	1.45 ± 0.06 ^b^	19.93 ± 0.25 ^c^	2.31 ± 0.02 ^a^	87.42 ± 1.12 ^a^	238.63 ± 4.12 ^a^
M.C.C.1	0.46 ± 0.02 ^e^	21.13 ± 0.58 ^c^	0.47 ± 0.02 ^ef^	41.64 ± 0.16 ^c^	79.08 ± 2.34 ^h^
M.C.C.2	0.46 ± 0.01 ^e^	22.34 ± 0.99 ^c^	0.41 ± 0.02 ^f^	28.46 ± 0.53 ^e^	75.06 ± 2.32 ^h^
M.C.C.3	0.79 ± 0.04 ^d^	34.03 ± 0.49 ^a^	0.55 ± 0.00 ^ef^	32.60 ± 0.23 ^de^	91.39 ± 2.16 ^g^
M.G.S.1	1.03 ± 0.03 ^c^	28.81 ± 2.11 ^b^	0.77 ± 0.03 ^d^	46.52 ± 2.18 ^c^	141.34 ± 8.38 ^d^
M.G.S.2	1.59 ± 0.06 ^b^	27.72 ± 0.52 ^b^	0.91 ± 0.02 ^cd^	37.56 ± 2.15 ^cd^	173.88 ± 5.04 ^b^
M.G.S.3	1.99 ± 0.08 ^a^	28.64 ± 1.68 ^b^	1.28 ± 0.09 ^b^	27.42 ± 1.62 ^e^	153.59 ± 1.20 ^c^

Values from the same column that do not share a letter are statistically different at *p* < 0.05 based on the Tukey method and 95% confidence. n = 3; M.G.I.—*Mytilus galloprovincialis* from Italy; M.C.C.—*Mytilus chilensis* from Chile; M.G.S.—*Mytilus galloprovincialis* from Spain; DW—dry weight.

## Data Availability

Data will be made available upon request.
